# Prevalence of methicillin sensitive and resistant Staphylococcus aureus carriage among German emergency medical providers

**DOI:** 10.3205/dgkh000490

**Published:** 2024-06-21

**Authors:** Aaron Weiss, Axel Kramer, Robert Taube, Frauke Mattner, Katrin Premke

**Affiliations:** 1 Institute of Hygiene and Environmental Medicine, University Medicine Greifswald, Greifswald, Germany; 2 Technical Microbiology, Faculty of Nature and Engineering, City University of Applied Sciences, Bremen, Germany; 3 Institute for Hygiene, Cologne Merheim Medical Centre, University Hospital Witten/Herdecke, Germany; 4 Department of Tropical Medicine and Infectious Diseases, Rostock University Medical Center, Rostock, Germany

**Keywords:** S. aureus carrier, MRSA carrier, nasal and throat carriage, emergency medical services, personal hygiene

## Abstract

**Background:**

Health care workers (HCW) in Emergency Medical Services (EMS) frequently come into contact with carriers of methicillin-susceptible *Staphylococcus* *aureus* (MSSA) and methicillin-resistant *Staphylococcus aureus* (MRSA) strains and may acquire and transmit them to patients. However, there is little data on MSSA and MRSA colonization of medical personnel in the emergency services. Additionally, few studies have analyzed the association between personal hygiene of staff and colonization. Therefore, we examined the prevalence of MSSA and MRSA in EMS staff of two German regions and evaluated their personal hygiene behavior.

**Method:**

Throat and nasal swabs from 300 EMS workers were analyzed. Both direct and pre-enriched cultures of the swabs were cultivated on culture media to identify MSSA and MRSA. Results were analyzed together with questionnaires about sociodemographic data and a self-assessment of hygiene behavior. Statistical analysis was done using the R statistical software.

**Results:**

Of the total 300 swabs, 55% were from paramedics, 39% were from emergency medical technicians (EMT) and 5% were from emergency physicians. With 1%, the MRSA prevalence was comparable to that of the German population, while the MSSA rate – 43.7% – was higher than expected. Colonization with MSSA was significantly associated with poor hand hygiene and male sex, and was inversely correlated to time on the job in EMS.

**Conclusion:**

The sample size of 300 and a MRSA prevalence of 1% made a meaningful analysis of potential influencing factors on the prevalence of MRSA infeasible. The comparatively high prevalence of MSSA and the association with decreasing frequency of hand antisepsis suggests an influence of personal hygiene on MSSA colonization. HCW in EMS should be encouraged to make use of their personal protective equipment and practice frequent hand hygiene. The implementation of diagnostic tools such as the Hand Hygiene Self-Assessment Framework of the WHO could be utilized to reveal problems in organizations, followed by an individual program to promote hand hygiene.

## Zusammenfassung

**Hintergrund:**

: Beschäftigte in Rettungsdiensten und Krankentransporten haben häufig mit Trägern von Methicillin-empfindlichen *Staphylococcus* *aureus*-Stämmen (MSSA) und Methicillin-resistenten *Staphylococcus* *aureus*-Stämmen (MRSA) Kontakt, können diese erwerben und auf Patienten übertragen. Allerdings gibt es nur wenige Daten über die MSSA- und MRSA-Kolonisierung von medizinischem Personal im Rettungsdienst. Darüber hinaus haben nur wenige Studien den Zusammenhang zwischen der persönlichen Hygiene des Personals und der Kolonisierung mit MSSA bzw. MRSA analysiert. Daher wurde die Prävalenz von MSSA und MRSA bei Rettungsdienstpersonal in zwei deutschen Regionen untersucht und ihr persönliches Hygieneverhalten bewertet.

**Methode:**

Es wurden Rachen- und Nasenabstriche von 300 Mitarbeitern von Rettungsdiensten untersucht. Sowohl direkte als auch angereicherte Kulturen der Abstriche wurden auf Nährböden kultiviert, um MSSA und MRSA zu identifizieren. Die Ergebnisse wurden zusammen mit Fragebögen zu soziodemografischen Daten und einer Selbsteinschätzung des Hygieneverhaltens ausgewertet. Die statistische Analyse wurde mit der Statistiksoftware R (Version 4.1.3) durchgeführt.

**Ergebnisse:**

Von den 300 Abstrichen stammten 55% von Notfallsanitätern, 39% von Rettungssanitätern und 5% von Notärzten. Die MRSA-Prävalenz war mit 1% vergleichbar mit der der deutschen Bevölkerung, während die MSSA-Rate mit 43,7% höher war als erwartet. Die Besiedlung mit MSSA stand in signifikantem Zusammenhang mit schlechter Händehygiene, männlichem Geschlecht und umgekehrt mit der Dauer der Tätigkeit im Rettungsdienst.

**Schlussfolgerung:**

Die Stichprobengröße von 300 und eine MRSA-Prävalenz von 1% erlauben keine aussagekräftige Analyse potenzieller Einflussfaktoren auf die MRSA-Prävalenz. Die vergleichsweise hohe Prävalenz von MSSA und der Zusammenhang mit der abnehmenden Häufigkeit der Händedesinfektion lässt auf einen Einfluss der persönlichen Hygiene auf die MSSA-Kolonisierung schließen. Die Mitarbeiter des Gesundheitswesens im Rettungsdienst sollten dazu angehalten werden, ihre persönliche Schutzausrüstung zu benutzen und die Indikationen der Händedesinfektion einzuhalten. Die Anwendung von Diagnoseinstrumenten wie dem Händehygiene-Selbstbewertungsrahmen der WHO könnte genutzt werden, um Probleme in Organisationen aufzudecken, gefolgt von einem individuellen Programm zur Förderung der Händehygiene.

## Introduction

Methicillin sensitive *Staphylococcus aureus* (MSSA) is an opportunistic pathogen which can colonize the upper respiratory tract. Cross-sectional studies have found an MSSA colonization rate of 20–30% of the population worldwide [1], [2] and 22–41% in the German population [3], [4], [5]. Compared to MSSA, colonization with MRSA is less common. with a prevalence ranging from 0.3–1.3% in the German population [3], [4]. Colonized humans are asymptomatic, with symptoms arising only in the event of infection. Thus, both MSSA and MRSA could unknowingly be spread within communities. Despite the low prevalence of MRSA in Germany, colonization with MRSA facilitates transmission and predisposes to various invasive infections [6], [7]. Both community acquired and nosocomial MRSA infections cause financial burdens [8], [9] and are responsible for an increased mortality [10]. The spread of MRSA is facilitated by direct contact to patients or healthcare workers (HCW) colonized or infected with MRSA [11], and can occur via airborne transmission in individuals with nasal carriage [12] as well as through contact to contaminated objects and surfaces on which MRSA can persist for months [13]. To minimize its spread, data on the frequency of MRSA carriage in HCW is needed. As emergency medical service (EMS) personnel could well play a unique role in transmission by linking the general population to high risk environments such as intensive care units (ICU), emergency departments (ED) and nursing homes [14], surveillance in this sector is important. Although there is a paucity of data on the prevalence of MRSA carriage in EMS [15], [16], it has been shown to vary from 0.7% to 21.2% [15], [16], [17], [18]. To reveal areas of high prevalence and contain transmission, local surveillance appears necessary. Furthermore, by evaluating the carrier rate of MRSA and MSSA in EMS personnel and comparing it to the general population, the efficacy of current infection control concepts can be assessed. It may be hypothesized that if the carrier rate in EMS, despite the higher risk of exposure [15], [18], is not significantly higher than the carrier rate of the general public, the protective measures in place are effective; this has already been confirmed in dental healthcare workers [19]. Thus, the aim of this study was to identify the prevalence of MSSA and MRSA and evaluate current infection control concepts of EMS in two geographically distant regions of Germany. 

## Materials and methods

### Study design and recruitment

The study performed in two different regions of Germany, Cologne and Greifswald. Throat and nasal swabs were taken from 304 EMS workers, which included emergency physicians (EP), emergency medical technicians (EMT) and paramedics from both fire departments and private EMS companies. Inclusion criteria were a minimum age of 18 years, voluntary written consent, a minimum work experience of one year and average weekly working time in EMS of at least 12 hours. Due to missing questionnaires, 300 swabs were analyzed. It is noteworthy that samples in the second cohort (Greifswald) were taken from 08.12.2020 to 30.06.2021 during peaks of the COVID-19 pandemic, while samples from the Cologne group were taken from 26.06.2019 to 07.10.2019.

### Sample collection and supporting questionnaire

To ensure proper sampling and minimize subjective error, the same person – who was instructed and trained in the swabbing technique – took samples from participants. A single swab was used to take samples from the posterior pharynx as well as from both anterior nares.

Additionally, participants filled out a questionnaire about demographics, weekly working hours, and years of experience. The questionnaire also contained items on self-assessment of the participants’ own hygiene behavior during different scenarios at work, treatment with antibiotics within the last two months, and the presence of immunodeficiency. Furthermore, contact to groups at risk for MSSA/MRSA colonization within the preceding six months were queried. Participants had to indicate whether they consciously had direct contact to those groups, e.g., in patient care/transport, or only indirect contact via co-workers or partners who cared for patients in the respective groups. Participants who stated that they had neither direct nor indirect contact were placed in the category of indirect contact, as we found it extremely unlikely that workers in EMS have no contact whatsoever with the groups in question. The complete questionnaire can be found in the supplements.

### Sample processing

Analysis of specimens was started within 48 hours after they were obtained. Swabs were transferred promptly to either the central laboratory of the Cologne-Merheim Clinic or the University Hospital Greifswald. Samples in the Cologne group were obtained from 26.06.2019 to 07.10.2019 using the Copan eSwab^®^ system (Copan, Italy). Samples from Greifswald were taken from 08.12.2020 to 30.6.2021 using Transystem^®^ with Amies agar gel medium (HAIN Lifescience, Nehren, Germany) [19]. Both direct and pre-enriched cultures were cultivated. Samples from the Cologne group were streaked onto Columbia CNA agar with 5% sheep blood to identify MSSA, while in the Greifswald group, mannitol salt agar (BD GmbH, Heidelberg, Germany) for MSSA was used. CHROMagar™ (BD GmbH, Heidelberg, Germany) was used in both groups to test for MRSA.

A detailed description of the methods can be found in the supplements. 

### Data preparation

Persons with missing answers or the answer “unsure” regarding contact to risk groups were excluded from the statistical analysis. The variables “soft tissue defects or chronic wounds” and “atopic dermatitis” were not analyzed, as they had a high number of missing answers which would have led to a drastic reduction of our datasets. After exclusion of those subjects, 262 datasets remained for analysis. Contact to persons working on ICU and patients receiving mechanical ventilation were placed in the cluster “ICU2”.

The cluster “Nursing” contained individuals working in outpatient care, individuals chronically in need of care, and individuals in long-term care facilities.

### Statistical analysis

Statistical analyses were performed with R software (version 4.1.3, R Foundation, Vienna, Austria). Statistical significance was set at p<0.05. The Chi-squared goodness-of-fit test was applied to compare the observed frequencies of infected and non-infected individuals to previous studies. Fisher’s exact test was employed to analyze pairwise dependence between variables. The Bonferroni method was used to correct for multiple comparisons. The Wilcoxon rank-sum test was performed to analyze differences between two groups. Spearman’s correlation was used to analyze associations between variables with multiple levels. Multivariate logistic regression models (binomial distribution) were applied to analyze the dependencies of replies in the questionnaire on the state of colonization. Backward selection based on Akaike’s information criterion was conducted to select predictive variables. The final model was cross-validated with the caret package (version 6.0-9.4), using 20% of the dataset as test data with 100 resamplings.

## Results

### Baseline data

Out of 305 enrolled participants, 304 nasal swabs could be analyzed, 300 persons filled out the questionnaire and 262 datasets remained for analysis after adjustment of data. 

Participants from Greifswald were on average 7 years older than participants from Cologne (30 vs. 37 years, on average), with more people working 48-hour weeks (64.4% vs 45.2%, on average), and more experienced personnel, with 37.3% working >10 years in EMS compared to 21.2% in Cologne (Table 1 (Tab. 1)). Both groups contained four times more male (79%) than female participants (21%). Most participants were paramedics (55%), followed by emergency medical technicians (39%) and emergency physicians (5%). In terms of age distribution by professional group, physicians were mostly older persons, most paramedics were in their mid-20s to mid-30s, and EMTs tended to be under 25 years of age. In total, 60% of the participants had work experience of 6–25 years and worked on average 48 hours a week. Most of the participants were Caucasian (78%), 2% were Asian and 20% chose “other” or provided no information about their ethnicity. 

### MSSA and MRSA

Almost half of the total samples were positive for MSSA (43.7%) and 3 (1%) were MRSA-positive. MRSA carriage only occurred in the participants from Cologne, with the positively screened persons being distributed across the three professional groups. 

### Hygiene behavior

Table 2 (Tab. 2) shows data of the participants’ self-assessment regarding their personal hygiene behavior. 83.3% of participants stated they performed hand antisepsis after every treatment; 85% reported glove use during every treatment. Use of facemasks, gowns and goggles differed between the two cohorts, with participants from Greifswald displaying more frequent use of this personal protective equipment (PPE). The most pronounced difference between the two cohorts was the use of facemasks: in Cologne, 0.4% stated wearing one during every call, but in Greifswald, 89.8% of the participants claimed they used facemasks for each treatment (p<0.0001). Hygiene behavior showed some statistically significant differences according to duration of employment in EMS. Participants with longer employment in EMS showed a reduced use of gloves (ρ=–0.21, p.adj=0.0006), but a significantly increased use of facemasks (ρ=0.16, p.adj=0.009), while hand antisepsis remained similar (ρ=–0.02, p.adj= 0.71).

### Probability of colonization with MSSA

**Figure 1:**
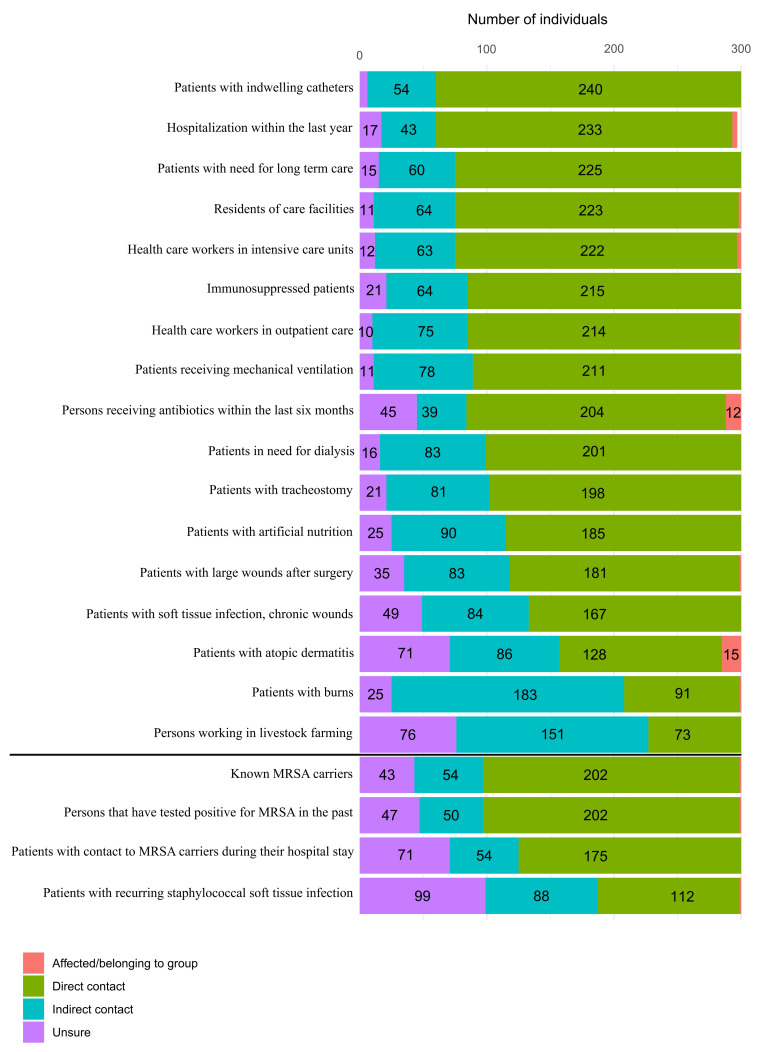
Contact of participants to groups at risk for MSSA/MRSA colonization. Participants had to specify whether they received antibiotics within the last two months, if they were diagnosed with an immune deficiency and whether they consciously had direct contact to the above-mentioned groups, such as in patient care/transport, or only indirect contact, such as via co-workers or partners who cared for patients in the respective groups. People stating they had neither direct nor indirect contact were placed in the category of indirect contact.

Through backward selection, we identified several variables that predicted MSSA colonization (accuracy after cross validation=0.64; McFadden R^2^=0.24). Gender, duration of employment, hospitalization, and hand hygiene were significant predictors. Male individuals showed a significantly higher probability of being colonized with MSSA (p=0.007). Our data showed that with increasing time on the job, it became less likely for persons to be colonized with MSSA (p=0.006). Especially people who had worked in EMS for over 10 years were less often colonized with MSSA. Concerning contact to risk groups, direct contact to persons who were hospitalized for more than three days within the last twelve months was highly significantly associated with MSSA colonization (p=0.005), whereas contact to persons with immunosuppression or the ICU2-cluster did not increase the likelihood of MSSA colonization (Figure 1 (Fig. 1)). Lastly, the more often people reported practicing hand hygiene, the less often they were colonized with MSSA (p=0.019).

## Discussion

To the best of our knowledge, this is the largest study testing for nasal/pharyngeal MRSA and MSSA carriage in EMS with additional consideration and evaluation of personal hygiene. Generally, studies on nasal *S*. *aureus* carriage in EMS are rare, although this profession is thought to be an important link between patients, hospitals and multidisciplinary staff [14]. 

### Method 

Studies examining MRSA prevalence in EMS often test for MRSA using swabs of the anterior nares [15], [16]. Although traditionally thought to be the most important site to test for S. aureus colonization [7], The sensitivity was shown to be as low as 48% [20] to 66% [21] when testing the nasal vestibule alone. We used one swab to test both the pharynx and both anterior nares, which in one study increased sensitivity by 25.7% [22]. Sampling by the same investigator guarantees a high level of standardization. Furthermore, the different analytical methods in laboratories influence the sensitivity in MRSA detection. One study showed that up to 1/3 of MRSA cases were overlooked when a direct culture without enrichment was used, compared to PCR analysis of the same sample [23]. We analyzed specimens using pre-enriched cultures, which exhibited a sensitivity only slightly lower than PCR analysis while being less cost-intensive [23].

### MRSA and MSSA nasal colonization in EMS 

With 1%, the prevalence of MRSA does not differ significantly from MRSA prevalence in the German general population (Table 3 (Tab. 3)). Regarding other countries, the prevalence among HCW of EMS in our study is in the range of the 0.7% reported for Virginia, USA [16], but lower than the 3.2% in 2018 in Hamburg, Germany [24], 4.6% in Ohio, USA [25] or 21.2% in Portugal (Table 4 (Tab. 4), [16], [17]). These geographical differences are well-known and partly attributable to misuse of antibiotics; however, the situation is much more complex due to geographical and socioeconomic factors, such as political stability, water quality, access to health care, and implementation of infection and surveillance control mechanisms [26], [27]. MSSA, on the other hand, shows a more even distribution of nasal colonization, with 20–30% globally and 22–41% in the German population [1], [2], [3], [4]. The MSSA rate of EMS in Greifswald was 47.5%. This is twice as high as rates found in surveillance data in Northern Germany of the regional community with 21.9% [5] and 27.2% [3] and data of dental healthcare workers from Northern Germany (22.3%) [19]. This may indicate inconsistent adherence of HCW in EMS to their PPE and standard precautions. Due to the lack of surveillance data for MSSA in Cologne, the MSSA rate of 42.7% in EMS in Cologne could not be compared. However, the higher prevalence of MSSA in Greifswald than in Cologne (47.5% vs. 42.7%) was not statistically significant (p=0.72).

Additionally, we determined high rates of direct contact to patient populations that pose a risk for MSSA transmission (Figure 1 (Fig. 1)), as well as frequent contact with hospital environments, which could contribute to the higher rates of MSSA. Other factors, such as gender or ethnicity, may also explain the higher carrier rates in our study compared to the German general population, as it was shown that Caucasian people and men have higher rates of MSSA carriage [1]. Our study participants consisted of roughly 80% men and only 20% women, with 78% Caucasian ethnicity, which might drive up the prevalence of MSSA in this study. Most other studies in EMS differ regarding MRSA and MSSA distribution (Table 4 (Tab. 4)). Only one study showed similar distribution and prevalence, with 1.9% MRSA-positive and 57.7% MSSA-positive (p=0.134, [28]). The high heterogeneity of MSSA and especially MRSA prevalence in the different studies is worth mentioning. These differences, with their poorly traceable and likely multifactorial influences, lead to the conclusion that each health care sector (EMS, ED, ICU) must undertake their own investigations to find out whether a serious burden of MSSA and MRSA exists. In doing so, further outbreaks could be prevented and sources could be isolated. 

Due to sampling during the COVID-19 pandemic in the second cohort (Greifswald), PPE use was enhanced by using FFP2 masks and safety goggles during every call without suspicion of COVID-19, and by using additional gowns/aprons around patients with suspected or confirmed COVID-19 infection. As such hygienic measures might also protect against colonization with different microorganisms, and our data show high adherence to use of PPE (Table 3 (Tab. 3)), the rate of MRSA carriage could have been reduced by this. 

### Distribution of nasal MRSA colonization and its limitations in evaluation

As previously stated, pronounced differences in prevalence are multifactorial, including differences in the prevalence of MRSA in the various geographic regions, local EMS protocols for hygienic measures and adherence of staff to them. Moreover, the type of study (longitudinal or cross-sectional) and testing influences results. Cross-sectional studies might fail to depict the true prevalence of MRSA, as colonization with *S. aureus* is not a dichotomous, but rather a dynamic event, including roughly 30% intermittent carriers which, in point-prevalence studies can either test positive or negative [1]. Although conducting longitudinal studies with a series of tests at different times can solve this problem, such studies are more difficult, complex, time consuming and cost intensive. While cross-sectional studies are not the most accurate indicator of MRSA carriage, they can provide an estimate to evaluate whether local hygiene protocols in place are effective in containing MRSA spread to protect healthcare workers and their patients. 

### Influence of socio-demographic characteristics on S. aureus carriage

Our data show that at the time of testing, the majority of EMS personnel in both cohorts was male (79%) and Caucasian (78%), which is in accordance with other studies [15], [25].

The significantly higher association of MSSA carriage with male gender is well recognized [3], [4], [29]. The reasons for this are manifold, including less optimal hand hygiene practiced by males [30], [31] and differences in sex hormones, which were shown to modulate immune response and interaction of host and microorganism, leaving males more susceptible to many bacterial infections [32], [33]. In both our cohorts, not older age itself but longer time in EMS was associated with statistically significant decreases in MSSA rates. Only a few studies investigating MSSA prevalence failed to demonstrate a significant correlation of age to MSSA carriage [5], [34], while most studies showed an inverse correlation of age with colonization status [1], [3], [29]. A possible explanation for this phenomenon could be an immunity buildup due to repeated exposure to *S. aureus*. Also, one could hypothesize that our study did not show significant age-related differences in MSSA carriage due to a lack of enough older participants to demonstrate the effect. Furthermore, a longer time in EMS could accelerate the natural immunity buildup due to more frequent exposure to MSSA and thus mimic the effects of older age on colonization status.

### Importance of the hygiene concept in the spread of S. aureus

Implementing systems to contain the spread of pathogens and especially MDRO (multidrug-resistant organisms) is the key element in reducing healthcare-associated infections. HCW are a recognized vector in transmitting MDRO [11], [35] and often show suboptimal compliance with standard precautions such as hand hygiene [36]. Especially EMS providers were found to have extremely low adherence to standard indications for practicing hand hygiene and standard hygiene precautions [37], [38]. Our questionnaire used basic scenarios (during every treatment, during most treatments, with suspected infection, never) from which participants could choose when they practice different precautions and use of PPE. As using the term “during every treatment” does not cover the different indications for practicing hand hygiene, one can only assume that the percentage of EMS workers correctly practicing hand hygiene is even lower. Several studies show that implementation of programs to increase hand hygiene compliance are effective, can reduce MRSA cross-transmission, nosocomial-/bloodstream infections with MRSA, and are cost-effective [39], [40]. Collection of data via questionnaires can help to reveal fundamental hygiene problems within organizations, but it does not replace studies that aim to assess compliance. The results regarding adherence to hygiene measures in this study can only indicate a trend. It is also worth mentioning that despite anonymization, the self-assessment might be susceptible to social desirability bias, where the participants tend to choose answers they know are favorable. In conclusion, it might be beneficial to err on the side of caution, assume knowledge gaps and poor compliance, and implement programs to prevent as many transmissions and infections as possible.

## Limitations

With a carrier rate for MRSA of 1%, the sample size of 300 does not allow any conclusions to be drawn about potential influences on colonization.

Another limitation is the possibility of selection bias, as known MRSA carriers might tend to not participate in this study, by which MRSA prevalence could be underestimated. In contrast to the low MRSA rate, the MSSA prevalence was 43.67%, which is noticeably high. This should be interpreted with the caveat that cross-sectional studies do not identify intermittent carriers, thus possibly over- or underestimating the true prevalence. Furthermore, as we used a self-assessment for hygiene behavior, the reported adherence may be overestimated.

## Conclusions

With a prevalence of 1%, MRSA prevalence in EMS does not differ from that of the German population. The high MSSA prevalence, the correlation of MSSA carriage with poor hand hygiene, and the known low adherence of EMS staff to the latter lead to the conclusion that there is room for improvement regarding hygienic behavior in German EMS staff. We recommend implementing a diagnostic tool such as the Hand Hygiene Self-Assessment Framework of the WHO [36] to reveal problems in hygiene, followed by an individual program to promote hand hygiene in the respective institutions.

## Notes

### Authors’ ORCIDs

- Aaron Weiss: 0009-0002-7735-7182
- Axel Kramer: 0000-0003-4193-2149
- Robert Taube: 0000-0003-3136-8732
- Frauke Mattner: 0009-0008-2268-6310
- Katrin Premke: 0000-0001-6216-5386

### Ethical approval

This study was conducted after approval by the ethics committee of the University of Greifswald (internal registration number BB042/19). 

### Funding

None.

### Acknowledgments

We gratefully acknowledge the participation of the 304 EMS workers for filling out the questionnaire and for allowing us to take the swabs. The project could not have been carried out without their help. We thank Ingo Winterfeld from the Institute for Hygiene of the Cologne-Merheim Clinic for helping us set up and organize sampling and analysis in Cologne.

### Competing interests

The authors declare that they have no competing interests.

## Figures and Tables

**Table 1:**
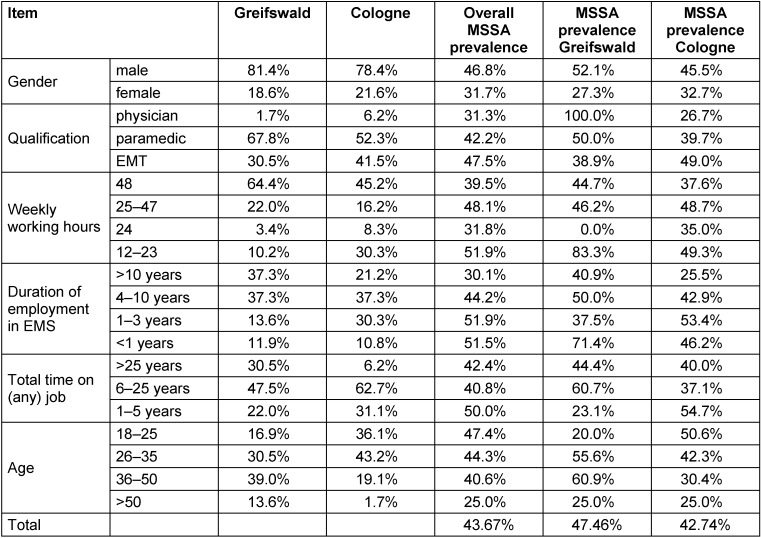
Sociodemographic differences in prevalence of MRSA and MSSA samples in Greifswald and Cologne

**Table 2:**
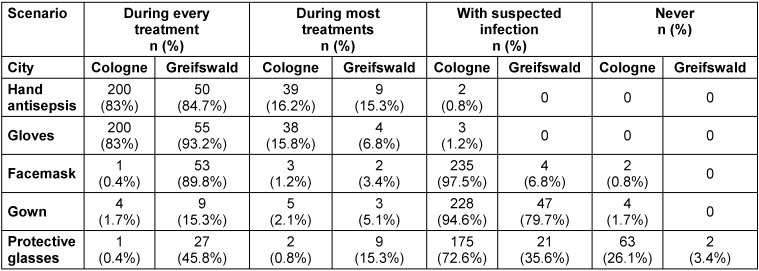
Data of the self-assessment of participants’ hygiene behavior during work

**Table 3:**
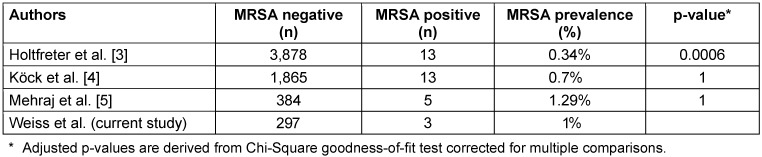
MRSA prevalence in the German population

**Table 4:**
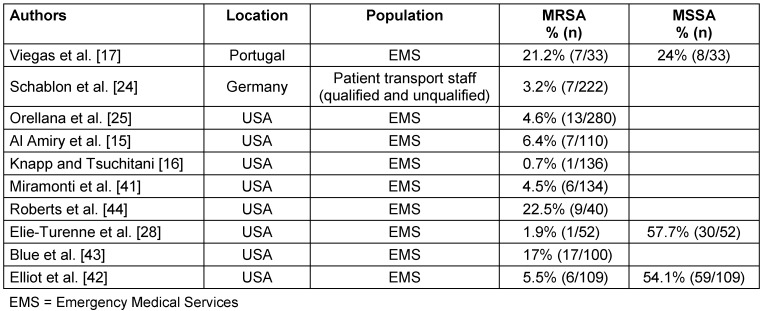
Prevalence of MRSA and MSSA carriages in different populations EMS
